# Immunogenic molecules associated with gut bacterial cell walls: chemical structures, immune-modulating functions, and mechanisms

**DOI:** 10.1093/procel/pwad016

**Published:** 2023-04-04

**Authors:** Ruopeng Yin, Tao Wang, Huanqin Dai, Junjie Han, Jingzu Sun, Ningning Liu, Wang Dong, Jin Zhong, Hongwei Liu

**Affiliations:** State Key Laboratory of Mycology, Institute of Microbiology, Chinese Academy of Sciences, Beijing 100101, China; Savaid Medical School, University of Chinese Academy of Sciences, Beijing 100049, China; State Key Laboratory of Mycology, Institute of Microbiology, Chinese Academy of Sciences, Beijing 100101, China; Savaid Medical School, University of Chinese Academy of Sciences, Beijing 100049, China; State Key Laboratory of Mycology, Institute of Microbiology, Chinese Academy of Sciences, Beijing 100101, China; Savaid Medical School, University of Chinese Academy of Sciences, Beijing 100049, China; State Key Laboratory of Mycology, Institute of Microbiology, Chinese Academy of Sciences, Beijing 100101, China; Savaid Medical School, University of Chinese Academy of Sciences, Beijing 100049, China; State Key Laboratory of Mycology, Institute of Microbiology, Chinese Academy of Sciences, Beijing 100101, China; Savaid Medical School, University of Chinese Academy of Sciences, Beijing 100049, China; CAS Key Laboratory of Pathogenic Microbiology and Immunology, Institute of Microbiology, Chinese Academy of Sciences, Beijing 100101, China; State Key Laboratory of Mycology, Institute of Microbiology, Chinese Academy of Sciences, Beijing 100101, China; Savaid Medical School, University of Chinese Academy of Sciences, Beijing 100049, China; State Key Laboratory of Microbial Resources, Institute of Microbiology, Chinese Academy of Sciences, 100101 Beijing, China; State Key Laboratory of Mycology, Institute of Microbiology, Chinese Academy of Sciences, Beijing 100101, China; Savaid Medical School, University of Chinese Academy of Sciences, Beijing 100049, China

**Keywords:** gut commensal bacteria, peptidoglycan, lipid-related molecules, immune responses

## Abstract

Interactions between gut microbiome and host immune system are fundamental to maintaining the intestinal mucosal barrier and homeostasis. At the host-gut microbiome interface, cell wall-derived molecules from gut commensal bacteria have been reported to play a pivotal role in training and remodeling host immune responses. In this article, we review gut bacterial cell wall-derived molecules with characterized chemical structures, including peptidoglycan and lipid-related molecules that impact host health and disease processes via regulating innate and adaptive immunity. Also, we aim to discuss the structures, immune responses, and underlying mechanisms of these immunogenic molecules. Based on current advances, we propose cell wall-derived components as important sources of medicinal agents for the treatment of infection and immune diseases.

## Graphical Abstract

**Graphical Abstract:**
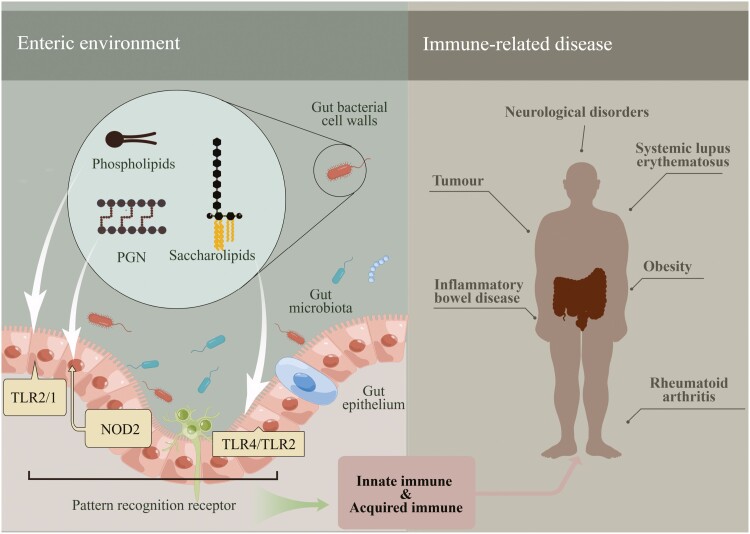

## Introduction

In the past decades, growing evidences have demonstrated that gut microbiota has profound influences on human health (Schroeder and Bäckhed, 2016; Krautkramer et al., 2021). The co-evolution of host and gut microbiome has led to a mutually beneficial ­consortium with a finely tuned immune system (Cebra, 1999). Gut microbiome and host immunity (especially gut mucosal immunity) interacts in a complex, dynamic, and environmentally dependent manner (Mallott and Amato, 2021). Alterations in gut microbiota have been identified in many immune-related diseases, such as inflammatory bowel disease (IBD), rheumatoid arthritis (RA), and systemic lupus erythematosus (SLE) (Yang and Cong, 2021; Jiang et al., 2022). It has been reported that the diversity of gut microbiome in IBD patients is decreased relative to the healthy subjects in the case cohort-based studies, with the phylum Firmicutes declining and Proteobacteria (especially Enterobacteriaceae) expanding (Jacobs et al., 2016; Sun et al., 2021). As to RA, the segmented filamentous bacteria was shown to trigger disease by inducing the differentiation and migration of intestinal T-follicle-assisted cells to the systemic lymphoid sites (Teng et al., 2016). On the other hand, the gut bacterium *Parabacteroides distasonis* was found to alleviate RA by suppressing T helper 17 (Th17) cells differentiation and promoting macrophage M2 polarization through bile acid metabolism (Sun et al., 2023). In SLE patient, the ratio of Firmicutes to Bacteroidetes in gut is significantly lower than that of healthy controls (Choi et al., 2020; Pan et al., 2021). In addition, the gut *Lactobacillus* have been reported to be associated with the remission of SLE (Zhang et al., 2014).

Gut microbiota is able to regulate intestinal innate and adaptive immune responses via immunorecognition receptors and immune cell populations [T cells, B cells, dendritic cells (DCs), and macrophages] (Alexander et al., 2014; Hou et al., 2022). The innate immune system which is the first line of gut defense against pathogenic infection maintains the intestinal homeostasis (Hoffmann et al., 1999). The pattern recognition receptors (PRRs) expressed in intestinal epithelial cells significantly affect the host innate immune responses by recruiting gut microbe-associated molecular patterns (MAMPs). To date, PRRs including toll-like receptors (TLRs), C-type lectin receptors, nucleotide-binding oligomerization domain (NOD)-like receptors (NLRs), retinoic acid-inducible gene-like receptors, absent in melanoma 2 (AIM2)-like receptors (ALR), and dectin1/2 receptors have been identified (Nenci et al., 2007; Palm and Medzhitov, 2009; Nigro et al., 2014). In the adaptive immune, Th17 cells that are abundant in the intestinal lamina propria were demonstrated preventing invasions of pathogens by expressing the retinoic acid receptor (RAR)-related orphan receptor (ROR)γ transcription factor and producing cytokines interleukin (IL)-17A, IL-17F, and IL-22 (Ivanov et al., 2006). These cytokines enable epithelial cells to generate antimicrobial peptides (AMPs), and thus enhancing the tight junctions (Ivanov et al., 2008; Weaver et al., 2013).

**Figure 1.**
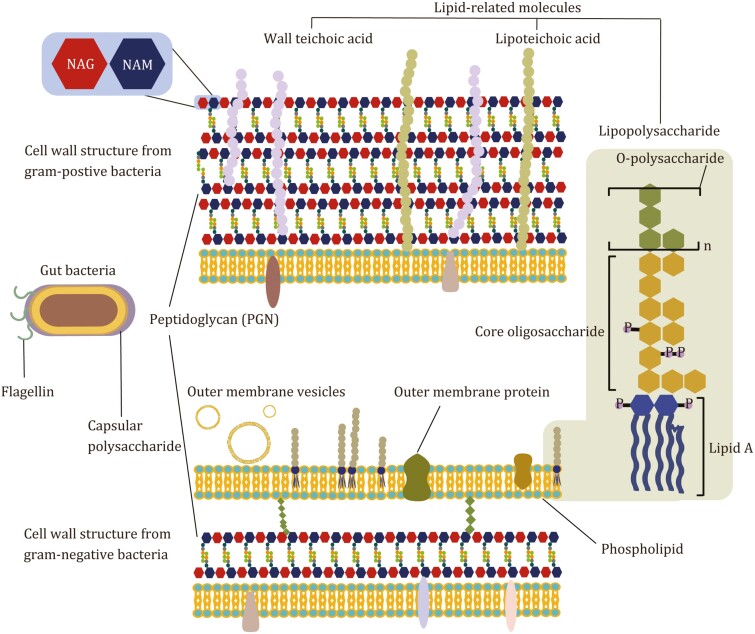
Cell wall-associated components of gut Gram-positive and Gram-negative bacteria.

Interactions between gut microbiome and host largely depend on microbial metabolites which are involved in signal transduction, regulation of metabolism and immune, and development of nervous system (Aron-Wisnewsky et al., 2021; Liu et al., 2021; Wu et al., 2021; Spindler et al., 2022; Chen et al., 2023). Notably, gut microbes-derived molecules that are constantly secreted or degraded in enteric cavity play critical roles in balancing the host immune response (Smith et al., 2013). Therefore, it is important to investigate the structural features of gut microbe-derived metabolites and their unique physiological activities. In this review, we summarize recent studies on gut bacterial cell wall-derived immunogenic molecules with characterized chemical structures, including peptidoglycan (PGN) and lipid-related molecules (Fig. 1) that shape innate and adaptive immune responses in the background of health and diseases.

## Gut microbes-derived immune-regulating molecules

**Figure 2.**
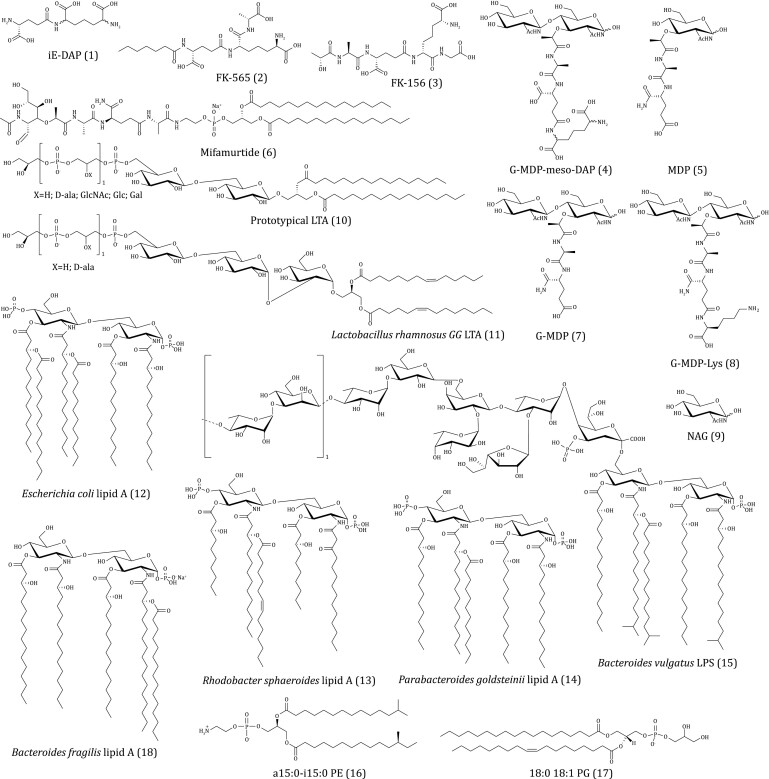
Representative structures of cell wall-derived immunogenic molecules (compounds 1–18) from gut microbiome.

To date, a close and inner linkage between gut microbiota and immunity homeostasis has been identified. However, the molecular mechanisms underlying interactions between gut microbiome and host immunity are not fully clarified. It is essential to elucidate the bacteria-derived signaling molecules and reveal their functions in conferring immune responses. Intestinal epithelia cells can directly interact with the cell wall-associated molecules of gut bacteria, sensing, and transducing signaling pathways to host immunity. Gut bacterial cell wall components are an important source of these immunogenic molecules, including PGN, lipopolysaccharide (LPS), lipoteichoic acid (LTA), phospholipids, etc. The structure of cell wall-associated molecules plays vital roles in addressing the immune-regulatory functions. Here, the representative structures of cell wall-derived immunogenic molecules from gut microbiome are outlined in Fig. 2.

### Production of PGN-derived immune-active molecules by gut microbes

Bacterial PGN that forms a multi-layer reticular macromolecular structure protects bacterial cells against the stress and maintains the tolerance of external environment (Egan et al., 2020). PGN is mainly composed of long polysaccharides chain with repetitive unit structure: N-acetylglucosamine (NAG) and N-acetyl muramic acid (NAM) (Fig. 1). The NAM is connected with an oligopeptide chain (four to five amino acids) that is further cross-linked with another NAM moiety on the second polysaccharide chains (Vollmer et al., 2008a).

**Figure 3.**
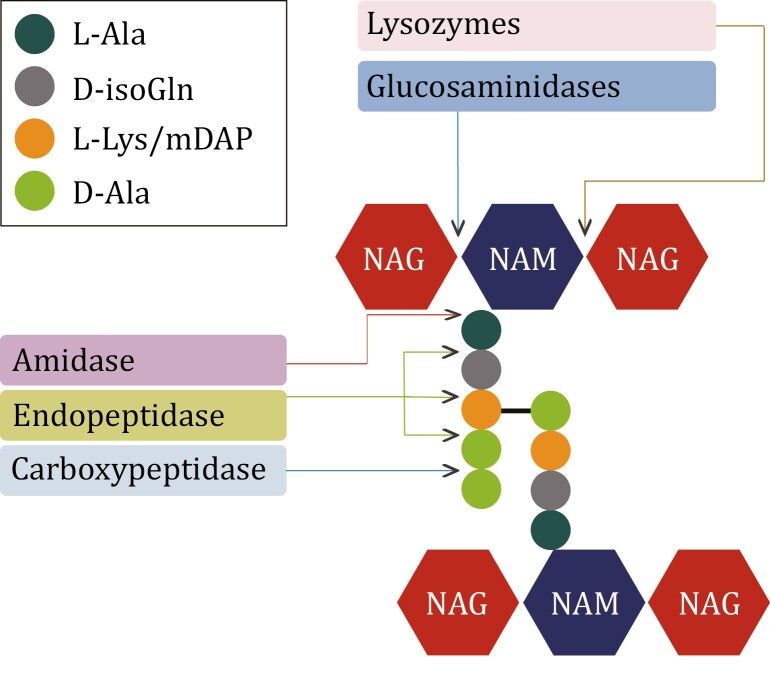
**The cross-linking peptidoglycan (PGN) molecule and the PGN hydrolases responsible for the lysis of PGN**. NAG, N-acetylglucosamine; NAM, N-acetylmuramic acid; mDAP, meso-diaminopimelic acid.

Bacterial PGNs are assembled by glycosyltransferase that polymerizes glycan chains and transpeptidase that catalyzes the peptide cross-linking (Typas et al., 2012). In addition, peptidoglycan hydrolases (PGHs) widely present in bacteria are responsible for the lysis of PGN (Vollmer et al., 2008b) (Fig. 3). The glycan chain of PGN contains two types of glycosidic bonds that are differentially sensitive to glycosidase activity. The β-1, 4-glycosidic bond between NAG and the adjacent NAM is hydrolyzed by N-acetyl-β-glucosaminidase, while the lysozymes cleavage the β-1,4-glycosidic bond between NAM and NAG residues by adding water in the glycosidic bond to produce NAM (Callewaert and Michiels, 2010; Egan et al., 2020). Amidase specifically hydrolyzes the amide bond between the first amino acid of the oligopeptide chain and NAM (Charroux et al., 2018). The peptidases are divided into two categories depending on the site of action: carboxypeptidases that remove the C-terminal amino acid of the oligopeptide and endopeptidases that cleft inside the peptide cross-linking. Peptidases are named as DD-, LD-, or DL-peptidases according to the isomers of the two amino acids to be cleaved (Vollmer et al., 2008b). Genes encoding DD-carboxypeptidase are widely distributed in all phyla of gut microbes, while the genes encoding DL-endopeptidases (especially the secreted type) are specifically encoded by gut bacteria in Firmicutes (Erysipelotrichaceae, Lachnospiraceae, and Ruminococcaceae) (Zou et al., 2019; Gao et al., 2022). A large amount of PGN fragments are dynamically and constantly generated from cell walls of the commensal bacteria in gut (Adam et al., 1981; Dworkin, 2014). To determine the level of PGN fragments in gut, approaches using the fluorescence imaging, the PGN-specific labeling probes with modified side chains of d-amino acids, and the monoclonal antibody that targets the conserved minimal immune-stimulatory structures of PGN were successfully developed (Huang et al., 2019; Brown et al., 2020; Wang et al., 2020c, Apostolos et al., 2022; Mondragón-Palomino et al., 2022). A recent study, a cell-based detection method using NOD1/2-transfected and nuclear factor kappa-B (NF-κB) luciferase-co-expressed HEK293T cells has been established. The intensity of fluorescence due to the activation of NOD1/2 pathway in the HEK293T cells corresponds to the level of the immune-active PGN molecules (Kim et al., 2019). In addition, the composition of PGN fragments can be studied *in vitro* by the paper chromatography, the thin layer chromatography, as well as the ultra-performance liquid chromatography with tandem mass spectrometric (UPLC-MS/MS) analysis (Ohya et al., 1993; Whitney et al., 2017). However, to further unveil the functions of PGN-related molecules in gut microbiota, new efficient analysis methods should be developed.

### Immunological functions of PGN-derived molecules

Previous studies have demonstrated that the soluble PGN-derived molecules from gut bacteria can act as immune-regulatory factors (Palm and Medzhitov, 2009). According to signaling pathways in immunity, the gut bacterial PGN-derived molecules are identified as activators of the NOD 1/2 receptors of cells. d-isoGlu-meso-diaminopimelic acid (iE-DAP, 1) from the PGNs of the gut commensal *Escherichia coli* and its chemical derivatives (Fig. 2) including heptanoyl-d-isoGlu-meso-DAP-d-Ala (FK-565, 2), d-lactoyl-l-Ala-d-isoGlu-meso-DAP-Gly (FK-156, 3), and an iE-DAP-derived tripeptide (NAG-NAM-L-Ala-d-isoGlu-meso-DAP, 4) have been confirmed as NOD1 agonists. Among these agonists, iE-DAP induces the secretion of IL-6 and tumor necrosis factor (TNF)-α by activation of NOD1 signaling pathway (Chamaillard et al., 2003), while its chemical derivatives FK-156 and FK-565 promote IL-1 secretion (Ahmed and Turk, 1989). FK-565 can promote colorectal cancer development by triggering the NOD1 pathway in tumor-infiltrating myeloid cells (Maisonneuve et al., 2021). iE-DAP-derived tripeptide (4) induces the production of IL-8 in epithelial cells by activation of NOD1/NF-κB pathway (Girardin et al., 2003). Further studies indicate that the meso-DAP containing in the structure of iE-DAP is necessary for the activation of NOD1 (Agnihotri et al., 2011). In addition, the gut bacteria-derived NOD1 activators in plasma contribute to the progression of metabolic inflammation and insulin resistance through production of macrophages-derived chemokine ligand (CXCL) 1 (Cavallari et al., 2017; Chan et al., 2017).

The activation of NOD2 by muramyl dipeptide (MDP, 5) derived from gut bacterial PGN is involved in anti-inflammatory events. It has been verified that the d-isoGln at the end of the peptide in MDP is essential for its specific activation of NOD2 pathway (Griffin et al., 2021). In addition, the phosphorylation of NAG of MDP *in vivo* by NAG kinase of hosts is necessary for its stimulant activity (Caruso et al., 2014; Stafford et al., 2022). In clinics, the muramyl tripeptide phosphatidyl ethanolamine (mifamurtide, 6) which is a derivative of MDP, acts as an immune modulator in the treatment of infections and cancers (Dvorožňáková et al., 2008). The underlying mechanism of actions for mifamurtide involves the activation of NOD2, which benefit for the gut mucosal immune homeostasis. In recent studies, several new MDP-related molecules with immune modulatory activities have been identified from gut commensal bacteria *Enterococcus faecium* and *Lactobacillus salivarius.* The gut commensal *E*. *faecium* shows protection against pathogen infections and enhances the anti-tumor efficacy of anti-programmed death (PD)-L1 in the melanoma model (Pedicord et al., 2016; Rangan et al., 2016), which is further demonstrated to rely on the expression an endopeptidase (the secreted antigen A, SagA) that cleaves the peptide bond of PGN. Furthermore, the activation of the MDP-regulated NOD2 pathway in the innate immune response is confirmed. The high-performance liquid chromatography with tandem mass spectrometric (HPLC-MS/MS) analysis indicates the presence of NAG-NAM-l-Ala-d-isoGln (G-MDP, 7) in the PGN fragments of gut *E*. *faecium* (Kim et al., 2019). Structural analysis of SagA revealed a NlpC/p60 family peptidase domain, in which the cysteine (C) 443A locus cleaves peptide bonds of d-isoGln and l-Lys to produce immunologically active PGN fragments. In the study of *L*. *salivarius*, a MDP analogue NAG-NAM-l-Ala-d-isoGln-l-Lys (G-MDP-Lys, 8) is isolated and demonstrated to alleviate the experimental colitis via activation of the NOD2-mediated signaling (Fernandez et al., 2011). Besides the immune-regulating function, MDP-activated NOD2 signaling is also implicated in the regulation of gut-brain crosstalk, development, and metabolism balance. It has been revealed that the NOD2-expressing GABAergic neurons are involved in the control of body temperature and appetite through sensing MDP from gut microbes (Gabanyi et al., 2022). PGN-derived molecules (MDP and its derivatives) from *Lactiplantibacillus plantarum*^WJL^ activate intestinal epithelial NOD2 receptor to promote insulin-like growth factor 1 production, contributing to the development of juvenile mice (Schwarzer et al., 2023). MDP and mifamurtide (6) mitigate obesity and insulin resistance via suppressing the interferon regulatory factor 4 (IRF4) due to activation of NOD2, whereas iE-DAP (1) and its derivatives exhibits the opposite effect (Cavallari et al., 2017).

Interestingly, new binding targets have been found for PGN and its fragments in recent works. On *Caenorhabditis elegans*, the muropeptide from *E*. *coli* in gut binds to and acts as an agonist of adenosine triphosphate (ATP) synthase, playing beneficial effects on mitochondrial homeostasis, development, and food behavior in animals (Tian and Han, 2022). The intact PGN (not fragments) from the gut *Lactobacillus* interacts with the extracellular receptor TLR2 to inhibit IL-12 production in macrophages (Shida et al., 2009). In another work, hexokinase that specifically binds with the PGN-derived NAG (9) can increase NLRP3 inflammasome in macrophages, suggesting it as a new innate immune receptor for recognizing (Wolf et al., 2016).

Early studies indicate rich endopeptidases with NlpC/p60 family peptidase-like domain in genomes of the human gut commensal bacteria (Zou et al., 2019; Gao et al., 2022). The HPLC-MS/MS analysis on muropeptides from PGN of gut commensal bacteria also reveals a variety of uncharacterized PGN-derived molecules. Based on these evidences, it can be deduced that the diverse PGHs encoded by gut commensal bacteria could produce a wide variety of PGN fragments. Above all, the structures and biological functions of these PGN-derived molecules need to be further explored.

### Immune functions of saccharolipids and phospholipids derived from bacterial cell wall

Gut bacterial cell wall-derived lipid-related molecules include saccharolipids and phospholipids. Saccharolipids from the cell walls of gut microbes include teichoic acid (TA) from Gram-positive bacteria and LPS from Gram-negative bacteria (Shiraishi et al., 2016) (Figs. 1 and 2). The wall-teichoic acid (WTA) not only plays a leading role in the attachment and colonization of commensals, but also contributes in part to the immune regulation of host (Kurokawa et al., 2013). The WAT derived from the intact cell wall of the gut probiotic *L*. *plantarum* stimulates IL-12 secretion from macrophages (Kojima et al., 2022). The LTA (10) from probiotics have shown beneficial effects on regulation of immune responses. In early works, LTA from the gut probiotic *L*. *rhamnosus* GG (LGGLTA, 11) counteracted ultraviolet (UV) B radiation-mediated immunosuppression and blocked tumor growth (Friedrich et al., 2019), which was ascribed to the increased migration of mesenchymal stem cell onto intestinal epithelial cells through TLR2-chemokine ligand (CXCL) 12 signaling pathway (Riehl et al., 2019); LTA of the probiotic *Apilactobacillus kosoi* stimulates the secretion of immune globulin A (IgA), neutralizing of toxins and pathogens (Matsuzaki et al., 2022); LTA of the gut commensal *Enterococcus faecalis* induces autophagy of macrophage cells via inhibiting phosphoinositid-3-kinase (PI3K)/protein kinase B (Akt)/mammalian target of rapamycin (mTOR) signaling pathway (Lin et al., 2018), and increases the inflammatory response through the production of TNF-α and IL-6 via p38 and extracellular regulated protein kinases (ERK1/2) signaling pathways in bone marrow-derived macrophages (Wang et al., 2019); LTA from the gut commensal *Lactobacillus paracasei* D3-5 effectively elevates the expression of mucin in mice by regulating TLR-2/p38-MAPK/NF-κB pathway, alleviating the age-related leaky gut and inflammation in mice (Wang et al., 2020b). TLR2 has been verified as the natural receptor of LTA, and activation of TLR2 signaling pathway facilitates secretion of mucin 2 on gut epithelia cells. Currently, only a few of gut bacteria-associated WTA and LTA have been studied for their effects on host. The functions of WTA or LTA from gut commensals with high abundance in the gut microbiota deserve much more attention and researches.

LPS released into circulation are known as potent inflammation inducers activating TLR4 or inflammatory caspase (caspase-4/5/11)-mediated signaling pathway (Rathinam et al., 2019; de Vos et al., 2022). In some recent works, LPS from gut commensals have been found to induce beneficial immune response in host. The suprarenal and celiac ganglia (SrG-CG) neurons can sense LPS from *E*. *coli* (Serotype O55:B5) to up-regulate the expression of neuropeptide Y (NPY) that reduce the splenic immune response and inhibit LPS-mediated inflammation (Yu et al., 2022). A penta-acylated LPS (lipid A moiety, 13) from the gut *Rhodobacter sphaeroides* corrects dysglycemia caused by hexa-acylated LPS (lipid A moiety, 12) from *E*. *coli* in lean mice and improves insulin sensitivity in obese mice (Anhê et al., 2021). Another LPS containing five acyl chains from the gut commensal *Parabacteroides goldsteinii* (lipid A moiety, 14) was deduced to alleviate chronic obstructive pulmonary disease (COPD) by inhibiting the TLR4 signaling pathway (Lai et al., 2022). The LPS derived from the gut *Bacteroides vulgatus* (BvLPS, 15) was reported to suppress inflammatory responses in IBD mice by inducing CD11c^+^ low-reactive semi-maturation of DCs (Steimle et al., 2019). As to its immunological properties, BvLPS induces a synergistic activation of the myeloid differentiation protein-2 (MD-2)/TLR4 and TLR-2-mediated pathway on human macrophages and DCs. The structure of BvLPS is established with five acyl chains, mono-phosphorylated lipid A, and a galactofuranose-containing oligosaccharide chain (Di Lorenzo et al., 2020). Furthermore, the underacylated LPS containing in members of gut order *Bacteroidales* is found to silence TLR4 signaling (d’Hennezel et al., 2017). Based on these findings, we deduce that the subtle structural variations in LPS from gut commensals lead to different immune-modulating activities. Gut microbes show great potential in biosynthesizing LPS with diverse structure and vital functions in modulating immune response. Much efforts should be made to study the chemical characteristics and bioactivities of these gut microbial LPS in the field of immunity, inflammation, and metabolism.

Phospholipids on bacterial membranes can serve as lipid antigens to regulate innate and adaptive immune response. Recently, the diacyl phosphatidylethanolamine (PE) with two branched chains (*anteiso*15:0-*iso*i15:0 PE, 16) was characterized from the gut commensal *Akkermansia muciniphila* and confirmed to agonize the TLR2/1 heterodimer signaling pathway inducing production of specific proinflammatory cytokines (Bae et al., 2022). It was also found that low doses of a15:0-i15:0 PE “passivate” the activation threshold of DCs and suppress the response to the subsequent LPS immune stimulation (Bae et al., 2022). The CD1 positive antigen-presenting cells have been demonstrated to recognize endogenous and microbial phospholipids and activate specific T cell populations (Van Rhijn et al., 2016; Shahine et al., 2019). In one study, the bacterial membrane phosphatidylglycerol (PG, 17) from *Staphylococcus aureus* in the gut microbiota and its chemically modified derivative (lysyl-PG) with a lysine head were verified to stimulate tetramer^+^ CD4^+^ T cell lines to secrete inflammatory cytokines of type 2 helper T cells (Monnot et al., 2023). In another work, the PE and phosphatidylcholine (PC) from the gut *Desulfovibrio piger* were found to promote gamma-delta T cell activation in the intestinal cells through CD1d, which further induced IL-17A production and exacerbated hypoxic-induced intestinal injury (Li et al., 2022). In some Gram-negative bacteria, the phospholipid-bound capsules polysaccharides (CPS) are present on the outer membranes (Willis et al., 2013). In the genome of the gut *Bacteroides fragile*, at least eight different CPS biosynthetic gene clusters are annotated, of which polysaccharide A (PSA) is most expressed one (Chatzidaki-Livanis et al., 2010). The PSA of *B*. *fragilis* binds TLR-2/1 heterodimer and Dectin-1 receptors to trigger PI3K/Akt signaling pathway on macrophages and DCs (Erturk-Hasdemir et al., 2019), and shows anti-inflammatory effect via secretion of IL-10 (Ramakrishna et al., 2019). Structure-activity relationship analysis indicates that the lipid of PSA (18) acts as an agonist for TLR-2/1 heterodimers, while the carbohydrate moieties of PSA bind to Dectin-1 (Erturk-Hasdemir et al., 2019). In the future work, the structural features of gut bacterial PE and PC with important immune-modulating activities should be examined to explore structure-activity relationship.

Lipid molecules with different chemical structures from gut bacteria can act as ligands for TLR receptors to modulate downstream immune responses (e.g., LPS for TLR4; LTA for TLR2; PE for TLR2/1). The binding site on the receptor and the recognition pattern between cell wall-derived lipid molecules and the TLR receptors need further investigation, which will enhance our understanding of mechanisms underlying the immune response. In addition, comparing to rich species diversity of gut bacteria, only a small number of lipid-related immunogenic molecules (Saccharolipids and phospholipids) from a few of gut bacteria have been studied, and many issues should be addressed before we can fully understand how lipid-related molecules in gut communicate with the immune system. The first issue is to establish the delicacy lipidomic profiles from all culturable gut microbes and characterize lipid-related immunogenic molecules by liquid chromatograph-mass spectrometric (LC-MS) or liquid chromatography-nuclear magnetic resonance (LC-NMR) analysis with the aid of artificial intelligence algorithms. The second issue is that currently reported immune-modulating effects of gut microbes-derived lipids are mainly obtained by *in vitro* studies. Taking the complex immune microenvironments and complicated immune regulations into consideration, the efficacies and the related immune-modulating mechanism of gut microbes-derived lipids needs to be further investigated in animal models for different diseases. Importantly, deep researches on lipid-related immunogenic molecules from gut bacterial will provide new strategy for treating infections and immune diseases, as exemplified by the immunoadjuvants developed from LPS (Di Lorenzo et al., 2019).

## Conclusions and perspectives

**Figure 4.**
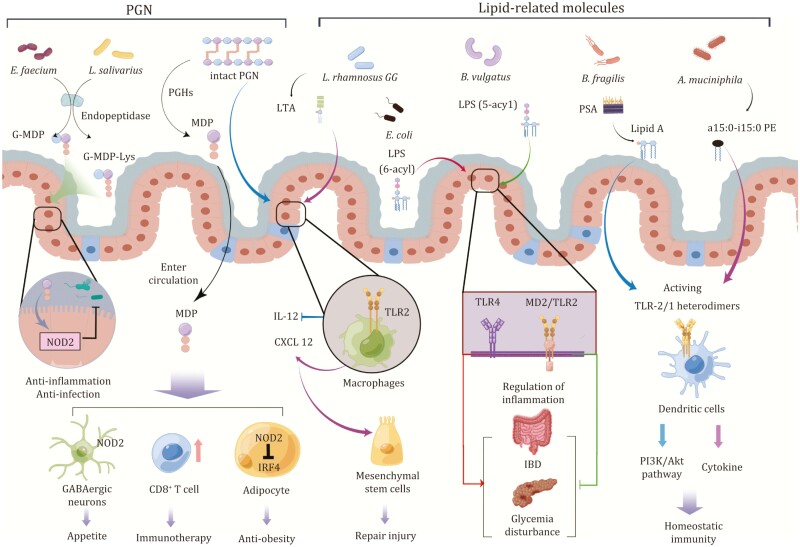
**Targets and signaling pathway modulated by gut microbiome-derived immunogenic molecules.** The production of G-MDP-Lys and G-MDP by *L*. *salivarius* and *E*. *faecium* depends on its endopeptidase activity. Both derivatives show anti-inflammatory or anti-infective activity in the intestinal epithelium by triggering NOD2. MDP enters the circulation, contributing to activation of the NOD2 pathway, which regulates appetite via GABAergic neurons; enhances the immunotherapy effect of tumor via CD8^+^ T cell expansion; reduces weight by inhibiting IRF4. Intact PGN could inhibit IL-12 production by acting on TLR2. For lipid-related molecules, LTA from LGG also stimulates CXCL12 production by binding to TLR2 of macrophages, thereby promoting mesenchymal stem cells to migrate to epithelium for injury repairment. The hexacylated LPS from *E*. *coli* is a potent agonist of TLR4, promoting colitis and metabolic disorders, while pentacylated LPS from *B*. *vulgatus* have anti-inflammatory activity dependent on MD2/TLR2 and TLR4. For lipids, lipid A from the PSA of *B*. *fragilis* triggers PI3K/Akt signaling via a heterodimer of TLR2/1, restoring immune homeostasis. The phospholipid antigen a15:0-i15:0 PE from *A*. *muciniphila* binds to the heterodimer of TLR2/1 to regulate the immune response of DCs. Figure 4 is drawn with Figdraw.

The interaction between the gut microbiome and host immune system has been extensively investigated in the past decades, accumulating much knowledge and insights underlying this important mutual relation (Fig. 4). In this paper, we review how gut microbial cell wall-derived immunogenic molecules including PGN and lipid-related molecules shape innate and adaptive immune responses during disease process. The PGN or PGN derivatives regulate host immune and inflammatory response via binding the intracellular innate immune receptors NOD1/2. The lipids of gut microbes can modulate the innate and adaptive immune response through regulation of the extracellular innate immune receptors-mediated pathway or specific T cell population. However, comparing to the diversity and complexity of gut microbiome, only a small number of gut commensals including *A*. *muciniphila*, *A*. *kosoi*, *B*. *fragilis*, *B*. *vulgatus*, *P*. *goldsteinii*, *E*. *coli*, *E*. *faecium*, *D*. *piger*, *L*. *paracasei*, *L*. *rhamnosus*, *L*. *casei*, *L*. *plantarum*, *L*. *salivarius*, *N*. *meningitides*, *R*. *sphaeroides*, and *S*. *aureus* have been explored for immune-active substances (Table 1). Besides immune-modulating molecules reviewed in this work, the cell wall-derived outer membrane (OM) proteins and outer membrane vesicles (OMV) also play regulatory roles in the cross-talking between gut microbiome and host immune system. For instance, The Amuc 1100, an OM protein of *A*. *muciniphila*, is demonstrated to improve gut barrier function and alleviate metabolic diseases, which could be related to its activation of the innate immune TLR2 signaling (Plovier et al., 2017). Amuc 1100 also improves colitis-associated colorectal cancer by reducing colorectal infiltrating macrophages and cytotoxic CD8^+^ T cells (Wang et al., 2020a). In a gut-brain axis study, another OM protein derived from *Escherichia coli*, OmpF/A, inhibits innate immune signaling through neuropeptides to manipulate digestion in the *C*. *elegans* (Geng et al., 2022). The OMV is a lipid-based delivery system for small molecules of the microbiome from bacteria to host cells. There is evidence that the lipids or encapsulated metabolites carried by OMV may contribute to many immunomodulatory properties. The OMV from gut *Bacteroides thetaiotaomicron* was reported to balance the regulatory DCs responses by stimulating IL-10 in the colonic DCs and IL-6 in the peripheral blood-derived DCs of healthy individuals (Durant et al., 2020). Much attentions and efforts should be paid to immunogenic molecules in the gut microbiota, with emphasis on their structures, immune-regulatory efficacy, and targeting signaling pathways. In addition, there are still large challenges to effectively detect these molecules in gut microbiota and accurately elucidate their structures. Further exploration of these gut microbes-derived immunogenic molecules will promote our understanding of new immune-regulatory mechanisms, which in turn sheds new light on developing clinical therapies for diseases.

**Table 1.** Immunogenic compounds derived from PGNs and lipids of gut microbes and their mechanisms of actions.

Cell wall-derived compounds	Origin	Mechanisms	Models investigated
iE-DAP	Synthesis	NOD1↑	Macrophages (Chamaillard et al., 2003)
FK-156	Synthesis	IL-1↑	Macrophages (Ahmed and Turk, 1989)
FK-565	Synthesis	NOD1↑	CRC mice (Maisonneuve et al., 2021)
G-MDP-meso-DAP	*N. meningitidis*	NOD1↑	NF-κB reporter HEK293T cells (Girardin et al., 2003)
Mifamurtide	Synthesis	NOD2↑	Clinical study (Dvorožňáková et al., 2008)
G-MDP	*E. faecium*	NOD2↑	Pathogen-infected mice (Kim et al., 2019)
G-MDP-Lys	*L. salivarius*	NOD2↑	IBD mice (Fernandez et al., 2011)
MDP	Synthesis	NOD2↑	Older mice (Gabanyi et al., 2022)
MDP	Synthesis	NOD2↑/IRF4↓	Obese mice (Cavallari et al., 2017)
Intact PGN	*Lactobacillus* spp.	TLR2↑	Peritoneal macrophages (Shida et al., 2009)
Muropeptides	*E. coli*	ATP synthase↑	Caenorhabditis elegans (Tian and Han, 2022)
NAG	*S. aureus*	Hexokinase↑	BMDMs (Wolf et al., 2016)
WTA	*L. plantarum*	IL-12↑	BMDMs (Kojima et al., 2022)
LTA	*L. rhamnosus* GG	TLR2↑	Radiation-injury (Riehl et al., 2019)
LTA	*L. paracasei* D3-5	TLR2↑	Obese mice (Wang et al., 2020b)
LTA	*A. kosoi*	lgA↑	Murine Peyer’s patch cells (Matsuzaki et al., 2022)
LTA	*E. faecalis*	PI3K/Akt/mTOR↓	RAW264.7 cell (Lin et al., 2018)
LTA	*E. faecalis*	p38/ERK1/2↑	BMDMs (Wang et al., 2019)
LPS	*E. coli*	TLR4↑	IBD mice (Heimesaat et al., 2007)
LPS	*P. goldsteinii*	TLR4↓	COPD mice (Lai et al., 2022)
LPS	*B. vulgatus*	MD-2/TLR4↑	IBD mice (Steimle et al., 2019; Di Lorenzo et al., 2020)
PSA	*B. fragilis*	TLR2/1 and Dectin-1↑	Macrophages (Erturk-Hasdemir et al., 2019)
a15:0-i15:0 PE	*A. muciniphila*	TLR2/1↑	DCs (Bae et al., 2022)
PG	*S. aureus*	CD4 + T cell↑	Atopic dermatitis (Monnot et al., 2023)
PE and PC	*D. piger*	γδ T cells↑	Hypoxia-injury (Li et al., 2022)

The importance of gut microbiome in remodeling immune responses has been widely recognized, only a small number of gut bacteria-derived immunogenic molecules have been studied. With the development of cultureomics methods and the optimization of anaerobic fermentation, more immunogenic molecules of gut commensals can be characterized. In addition, flow cytometry-click chemistry coupling methods could also enable us to identify gut bacterial community with specific cell wall-derived molecules (Tei and Baskin, 2022). Moreover, advances in bioinformatics analysis and genome manipulation realize the design and generation of live biotherapeutic products by expressing gut microbiome-derived immunogenic molecules in the probiotic strains including *E*. *coli* Nissle 1917 and *L*. *plantarum*. To increase our insights into the gut microbiome-host interaction, gene deletion and hetero-expression methods should be expanded to other important taxa including *Lactobacillus*, *Akkermansia*, and *Bifidobacteria* in human gut microbiome. Taken together, with more key functions of gut microbial cell wall-derived molecules being demonstrated, their biosynthesis, metabolisms, bioactivities, and mechanism of actions should be taken into consideration in studies of gut microbiota–host interactions.

## Abbreviations

Akt: protein kinase B
AMPs: antimicrobial peptides
ATP: adenosine triphosphate
CD: cluster of differentiation
COPD: chronic obstructive pulmonary disease
CPS: capsules polysaccharides
CXCL: chemokine ligand
DCs: dendritic cells
ERK: extracellular regulated protein kinases
GABA: γ-aminobutyric acid
G-MDP: NAG-NAM-L-Ala-D-isoGln
G-MDP-Lys: NAG-NAM-L-Ala-d-isoGln-l-Lys
HPLC-MS/MS: high-performance liquid chromatography with tandem mass spectrometry
IBD: inflammatory bowel disease
iE-DAP: d-isoGlu-meso-DAP
IgA: immune globulin A
IL: interleukin
IRF: interferon regulatory factor 4
LC-MS: liquid chromatograph-mass spectrometry
LC-NMR: liquid chromatography-nuclear magnetic resonance
LGG: Lactobacillus rhamnosus GG
LPS: lipopolysaccharide
LTA: lipoteichoic acid
MAMPs: microbe-associated molecular patterns
MD-2: myeloid differentiation protein-2
MDP: muramyl dipeptide
meso-DAP: meso-diaminopimelic acid
mTOR: mammalian target of rapamycin
NAG: N-acetylglucosamine
NAM: N-acetylmuramic acid
NF-κB: nuclear factor kappa-B
NLRs: NOD-like receptors
NOD: nucleotide-binding oligomerization domain
NPY: neuropeptide Y
OM: outer membrane
OMV: outer membrane vesicles
PC: phosphatidylcholine
PD-L1: programmed death-L1
PE: phosphatidylethanolamine
PG: phosphatidylglycerol
PGHs: peptidoglycan hydrolases
PGN: peptidoglycan
PI3K: phosphoinositid-3-kinase
PRRs: pattern recognition receptors
PSA: polysaccharide A
RA: rheumatoid arthritis
RORγt: RAR-related orphan receptor γ transcription factor
SagA: secreted antigen A
SLE: systemic lupus erythematosus
TA: teichoic acid
Th17: T helper 17
TLRs: toll-like receptors
TNF: tumor necrosis factor
UPLC-MS/MS: ultra-performance liquid chromatography with tandem mass spectrometry
UV: ultraviolet
WTA: wall-teichoic acid
